# Testicular sperm is superior to ejaculated sperm for ICSI in cryptozoospermia: An update systematic review and meta-analysis

**DOI:** 10.1038/s41598-018-26280-0

**Published:** 2018-05-18

**Authors:** Yi-No Kang, Ya-Wen Hsiao, Chien-Yu Chen, Chien-Chih Wu

**Affiliations:** 1 Centre for Evidence-Based Medicine, Department of Education, Taipei Medical University Hospital, Taipei, Taiwan; 2 School of Medicine, College of Medicine, Taipei Medical University, Taipei, Taiwan; 3 Department of Anesthesiology, Taipei Medical University Hospital, Taipei, Taiwan; 4 Department of Anesthesiology, School of Medicine, College of Medicine, Taipei Medical University, Taipei, Taiwan; 5 Department of Education in Medicine and Humanity, School of Medicine, College of Medicine, Taipei Medical University, Taipei, Taiwan; 6 Department of Urology, Taipei Medical University Hospital, Taipei, Taiwan; 7 Department of Urology, School of Medicine, College of Medicine, Taipei Medical University, Taipei, Taiwan

## Abstract

Intracytoplasmic sperm injection (ICSI) is well established and provides patients with severely impaired sperm quality with an opportunity to father a child. However, previous studies do not clearly indicate whether male with cryptozoospermia should use testicular sperm or ejaculated sperm for ICSI. The newest systematic review of this topic also gave a controversial conclusion that was based on incorrect pooling result. Moreover, two clinical studies published after the systematic review. In the present update systematic review and meta-analysis, a comprehensive citation search for relevant studies was performed using the Cochrane library databases, Embase, Ovid MEDLINE, PubMed, ScienceDirect, Scopus, and Web of Science up to September 2017. The search returned 313 records, in which six studies were included in quantitative synthesis. These studies involved 578 male infertility patients who had undergone 761 ICSI cycles. The risk ratios favour fresh testicular sperm for good quality embryo rate (1.17, 95% CI 1.05–1.30, *P* = 0.005), implantation rate (95% CI 1.02–2.26, *P* = 0.04), and pregnancy rate (RR = 1.74, 95% CI 1.20–2.52, *P* = 0.004). In conclusion, the existing evidence suggests that testicular sperm is better than ejaculated sperm for ICSI in male with cryptozoospermia.

## Introduction

Intracytoplasmic sperm injection (ICSI) is well established and provides hope for patients with extremely poor sperm quality, as in cryptozoospermia, to father children^1^. Sperm quality, including sperm count and motility, may influence the outcome of ICSI^2,3^. For a healthy fertile man with a normal sperm count and sperm motility, ejaculated sperm that have completed their maturation process in the male reproductive tract generally have a high fertilisation capability and can produce a natural pregnancy^4^. However, men with cryptozoospermia who have extremely low sperm count and sperm motility require ICSI. Cryptozoospermia is defined as the constant presence of isolated sperm cells in the ejaculate that can be detected after an extensive microscopic search^5^. The ejaculate collected from the patients with cryptozoospermia containing very few sperm cells, and these sperm cells commonly exhibit low motility. This kind of sperm may reduce ICSI success rate. Therefore, testicular sperm obtained through surgical retrieval for ICSI may be suggested^6–8^.

In these decades, whether testicular or ejaculated sperm produces better outcomes of ICSI, assessed as fertilisation rate, embryo quality, implantation rate, and pregnancy rate still remain controversial^6–11^. A recent systematic review and meta-analysis focusing on pregnancy rate and fertilisation rate concluded that evidence does not support the recommendation that patients with cryptozoospermia should prefer testicular sperm over ejaculated sperm for ICSI^12^. However, two studies^8,11^ including 340 patients with cryptozoospermia who had undergone 277 ICSI cycles have been published after the systematic review. Therefore, the purpose of this study was to perform a further examination on this issue.

## Results

### Evidence identified

**Figure 1:**
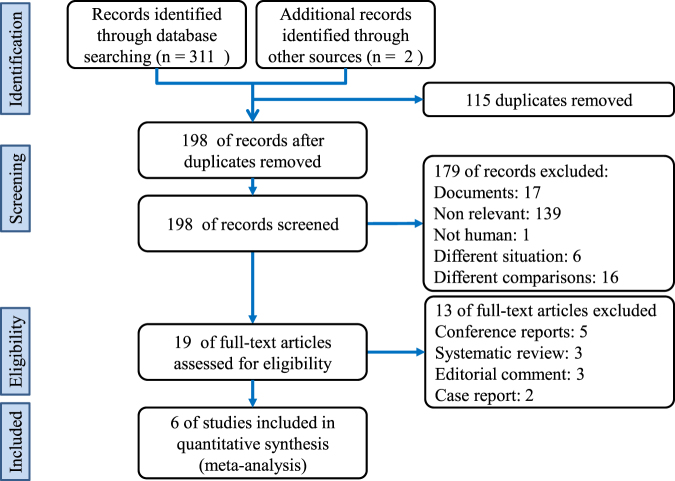
Diagram of this systematic review and meta-analysis according to the Preferred Reporting Items for Systematic Reviews and Meta-Analyses guidelines.

The search returned 313 citation records, of which 5, 53, 73, 25, 49, 70, and 36 citations were from the Cochrane library databases, Embase, Ovid MEDLINE, PubMed, ScienceDirect, Scopus, and Web of Science, respectively. The other two citations from the reference lists of manually screened relevant articles. Figure 1 depicts the processes of identification and selection of the studies. In total, 294 citations were excluded because 115 duplicates, 179 citations did not match the inclusion criteria of our study when the titles and abstracts were screened. After a full-text review of 19 citations, five conference reports, three systematic reviews, three editorial comments, and two case reports were excluded.

### Characteristics of the included evidence

The characteristics of the included studies are listed in Table 1. All six identified studies^6–11^ were cohort studies. These studies included 578 male infertility patients who had undergone 761 ICSI cycles including 541 ICSI cycles with ejaculated sperm, 153 ICSI cycles with fresh testicular sperm, and 67 ICSI cycles with frozen-thawed testicular sperm between 1993 and 2014. All studies except one^6^ reported isolated fresh testicular sperm data. In all the studies, testicular sperm was retrieved through microdissection testicular sperm extraction (mTESE)^9^, testicular sperm extraction (TESE)^6,7,10,11^, and TESE combined with testicular sperm aspiration (TESA)^8^. The other reported potential parameters were as follows: testicular size^10^, sperm conditions^7–9^, and DNA screening^7,10^. The characteristics and risk of bias summary of the included studies are presented in Table 1. In the six included studies, different ovarian hyperstimulation protocols were used. While four studies used long protocol with regime of gonadotropin-releasing hormone (GnRH) agonist and human menopausal gonadotrophin (HMG) or follicle-stimulating hormone (FSH)^6,8–10^, one study used modified super long protocol^11^ and the other one did not specify what protocol was used^7^. The further information of oocyte quality and assessment was performed in Supplementary Table 1. Individual assessment of risk of bias was presented in Supplementary Table 2.

**Table 1:** Characteristics and risk of bias of included studies.

Study & country	Study type	No. of patients	Included years	Mean paternal age (y)		Mean Maternal age (y)	
				Ejaculated	Testicular	Ejaculated	Testicular
Amirjannati *et al*.^9^ in Iran	Cohort study	192^a^	2005–2009	36.85 ± 5.34^e^	40.68 ± 4.94^e^	31.85 ± 5.76^e^	35 ± 5.91^e^
Ben-Ami *et al*.^6^ in Israel	Cohort study	17^a^	2010–2011	32.8 ± 5.3^e^	36.3 ± 6.6^d,e^	29.1 ± 3.4^e^	30.9 ± 4.1^d,e^
Bendikson *et al*.^7^ in USA	Cohort study	16^a^	1993–2005	36.7 ± 5^e^	37 ± 4^e^	31.6 ± 5^e^	32 ± 5^e^
Cui *et al*.^8^ in China	Cohort study	285^b^	2009–2013	34.24 ± 4.83^f^	35.12 ± 6.25^f^	28.15 ± 5.32^f^	29.31 ± 5.87^f^
Hauser *et al*.^10^ in Israel	Cohort study	13^a^	1996–2009	36.7 ± 8.3^c,e^		32.7 ± 4.5^e^	34.1 ± 5.1^d,e^
Ketabchi *et al*.^11^ in Iran	Prospective cohort study	55^b^	2011–2014	NR	NR	NR	NR
**Study & country**	**ICSI cycles**			**Method of surgical extraction**	**Other male parameters**	**Relevant outcomes**	**Quality of study**
	**Ejaculated(fresh)**	**Testicular(fresh)**	**Testicular(frozen)**				
Amirjannati *et al*.^9^ in Iran	208	19	0	mTESE	Sperm count, normal morpholoy and vitality	Fertilisation rate,Embryo quality	8 stars
Ben-Ami *et al*.^6^ in Israel	68	31	17	TESE	NR	Fertilisation rate,Pregnancy rate	8 stars
Bendikson *et al*.^7^ in USA	27	21	0	TESE	Genetic screening,sperm count	Fertilisation rate,Implantation rate,Pregnancy rate,	8 stars
Cui *et al*.^8^ in China	166	56	0	TESE or TESA	Testicular volume, sperm count, FSH level, BMI	Fertilisation rate, Embryo quality, Implantation rate, Pregnancy rate	9 stars
Hauser *et al*.^10^ in Israel	34	9	50	TESE	Genetic screening, FSH level, testicular size, testicular histology	Fertilisation rate,Embryo quality,Implantation rate,Pregnancy rate	9 stars
Ketabchi *et al*.^11^ in Iran	38	17	0	TESE	NR	Fertilisation rate,Embryo quality, Implantation rate, Pregnancy rate	7 stars

^a^Some patients underwent ICSI with both ejaculated sperm and testicular sperm. ^b^Ejaculated sperm group and testicular sperm group consisted of different patients. ^c^Mixed data of ejaculated sperm and testicular sperm groups. ^d^Mixed data of fresh testicular sperm group and frozen sperm groups. ^e^Mean ± standard deviation. ^f^Mean ± standard error. NR, not reported; FSH, follicle-stimulating hormone; BMI, body mass index; SDF, sperm DNA fragmentation; TESE, testicular sperm extraction; mTESE, microdissection TESE; TESA, testicular sperm aspiration.

### Primary outcomes

**Figure 2:**
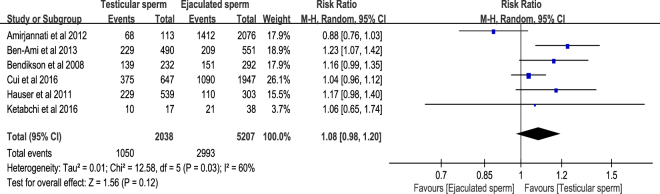
Forest plot of fertilisation rate between testicular and ejaculated sperm.

We defined the fertilisation rate as the number of normally fertilised ova (i.e. 2 pronuclei, 2PN) divided by the number of metaphase II oocytes. All the included studies^6–11^ reported the relevant information of fertilisation rate, in which 1050 fertilisations out of 2038 metaphase II oocytes in testicular sperm group and 2993 fertilisations out of 5207 metaphase II oocytes in ejaculated sperm group. The result of meta-analysis with random-effects model showed that the pooled risk ratio (RR) of fertilisation rate in testicular sperm group versus ejaculated sperm group was 1.08 with 95% confidence interval (95% CI) 0.98 to 1.32 (*P* = 0.12), thus indicating that testicular sperm had no significant fertilisation rate compared with ejaculated sperm for ICSI in male with cryptozoospermia. Moderate to high heterogeneity was detected in the meta-analysis of fertilisation rate (*I*^2^ = 60%, *P* = 0.03) (Fig. 2). The results had acceptable levels of publication bias that was detected by Egger’s test (*t* = 0.43, 95% CI −3.82–5.23, *P* = 0.69) and Begg’s test (т = −0.13, *P* = 0.71) (Supplementary Figure S1).

**Figure 3:**
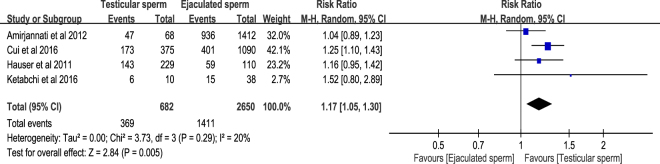
Forest plot of good-embryo rate between testicular and ejaculated sperm.

Embryo quality is determined by examining the size and symmetry of the blastomeres and assessing the fragmentation rate. Although “good-quality embryo” is defined as exhibiting <20% fragmentation in many studies^10,11^, Amirjannati *et al*. classified embryos with no fragmentation and 6–8 regular and symmetric blastomeres as grade A embryos^9^. With the aforementioned definition, the good-quality embryo rate as the number of good-quality embryos or grade A embryos divided by the number of fertilisation. Three of the included studies provided original data^8,10,11^, and the other one expressed as mean ± SD^9^. Therefore, we estimated good-quality embryo from mean ± SD that reported by Amirjannati *et al*.^9^. The included four cohort studies^8–11^ reported the relevant information of good-quality embryo rate, in which 369 good-quality embryos out of 682 embryos in testicular sperm group and 1411 good-quality embryos out of 2650 embryos in ejaculated sperm group. The pooled result in random-effects model revealed that testicular sperm had significantly higher good-embryo rate compared with ejaculated sperm for ICSI in patients with cryptozoospermia (RR = 1.17, 95% CI 1.05–1.30, *P* = 0.005). Low heterogeneity was detected in this meta-analysis (*I*^2^ = 20%, *P* = 0.29) (Fig. 3).

**Figure 4:**
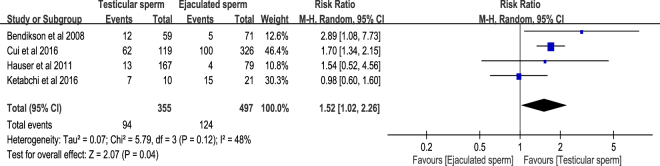
Forest plot of implantation rate between testicular and ejaculated sperm.

The definition of implantation rate in this systematic review was the number of gestational sacs seen on the initial examination divided by the number of embryo transfers (ETs) in all ICSI cycles with testicular sperm or ejaculated sperm. There were four identified cohort studies^7,8,10,11^ that presenting relevant information of implantation rate. The result of meta-analysis with random-effects model revealed that the RR of implantation rate in testicular sperm group versus ejaculated sperm group was 1.52 (95% CI 1.02–2.26, *P* = 0.04). This result indicated that testicular sperm had significantly higher implantation rate compared with ejaculated sperm for ICSI in patients with cryptozoospermia. Acceptable heterogeneity was found in the meta-analysis of implantation rate (*I*^2^ = 48%, *P* = 0.12) (Fig. 4).

**Figure 5:**
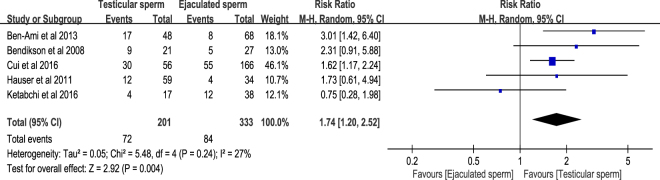
Forest plot of pregnancy rate between testicular and ejaculated sperm.

Pregnancy rate is expressed as the number of pregnancies divided by the number of ICSI cycles. Pooled result of pregnancy rate basis on the five included cohort studies^6–8,10,11^, in which ICSI cycles and pregnancies were reported. There were 72 pregnancies out of 201 ICSI cycles in testicular sperm group and 84 pregnancies out of 333 ICSI cycles in ejaculated sperm group. The pooled estimate with random-effects model indicated that testicular sperm had significantly higher pregnancy rate compared with ejaculated sperm for ICSI in patients with cryptozoospermia (RR = 1.74, 95% CI 1.20–2.52, *P* = 0.004). Acceptable heterogeneity was found in this meta-analysis (*I*^2^ = 27%, *P* = 0.24) (Fig. 5). The Egger’s test (*t* = 0.12, 95% CI −3.89–4.20, *P* = 0.91) and Begg’s test (т = −0.10, *P* = 0.81) showed no publication bias in this analysis (Supplementary Figure S2).

### Further analyses

Further analyses were conducted for giving more informative results in this systematic review and meta-analysis. The further analyses divided data into two subgroups according to data situation. One of subgroups conducted meta-analysis with fresh sperm data, and the other one of subgroups used estimated and mixed data that mixed frozen sperm data with fresh sperm data. To avoid double count data and overestimation, all the outcomes of subgroup analyses were reported as subtotal rather than total.

Regarding to subgroup analyses of fertilisation rate, four studies^7,8,10,11^ reported fresh sperm data and three studies^6,9,10^ reported estimated or mixed data. There were 1936 fertilisations and 3558 metaphase II oocytes in the four studies that reported fresh sperm data. There were 2217 fertilisations and 3990 metaphase II oocytes in the three studies that reported estimated or mixed data. Results of the subgroup analyses revealed that no significant differences between testicular sperm group and ejaculated sperm group in both subgroup of fresh sperm data (RR = 1.11, 95% CI 1.00–1.23, *P* = 0.06), and subgroup of frozen sperm and estimated data (RR = 1.07, 95% CI 0.87–1.32, *P* = 0.50). The heterogeneity in the subgroup of fresh sperm data was acceptable (*I*^2^ = 34%, *P* = 0.21), and it was high (*I*^2^ = 81%, *P* = 0.006) in the subgroup of frozen sperm data and estimated data (Supplementary Figure S3). Although the no statistical difference in fertilisation rates between the two groups, the trend favoured testicular sperm group especially using fresh sperm data. This trend may relate to DNA quality, because DNA quality in testicular sperm is better than in ejaculated sperm.

In subgroup analyses of good-embryo rate, three studies^8,10,11^ reported fresh sperm data and two studies^9,10^ reported estimated or mixed data. In fresh sperm data, there were 675 good-quality embryos out of 1663 embryos. In the estimated or mixed data, there were 1164 good-quality embryos out of 1779 embryos. Significant difference in good-embryo rate between testicular sperm group and ejaculated sperm group can be found in the subgroup of fresh sperm data (RR = 1.21, 95% CI 1.05–1.41, *P* = 0.01). However, pooled result in the subgroup of frozen sperm and estimated data showed that no difference between testicular sperm group and ejaculated sperm group (RR = 1.11, 95% CI 0.96–1.27, *P* = 0.16). Low heterogeneity was detected in both subgroups. Nevertheless, a trend can be seen whereby the heterogeneity in the subgroup analysis of fresh sperm data (*I*^2^ = 9%, *P* = 0.33) was lower than in the subgroup of frozen sperm data and estimated data (*I*^2^ = 17%, *P* = 0.27) (Supplementary Figure S4).

About subgroup analyses of implantation rate, four studies^7,8,10,11^ reported fresh sperm data and only one study^10^ reported data that including frozen sperm. There were 210 implantations out of 713 ETs in the four studies, and 12 implantations out of 931 ETs in the only one study that reported data including frozen sperm. Pooled estimate of implantation rate significantly favoured testicular sperm compared with ejaculated sperm in the subgroup of fresh sperm data (RR = 1.69, 95% CI 1.05–2.74, *P* = 0.03). The other subgroup showed no difference between the two sources of sperm (RR = 1.13, 95% CI 0.35–3.63, *P* = 0.84). Moderate to high heterogeneity was detected in the subgroup of fresh sperm data (*I*^2^ = 60%, *P* = 0.05) (Supplementary Figure S5).

Regarding to subgroup analysis of pregnancy rate, four studies^7,8,10,11^ reported relevant information with fresh sperm data and two studies^6,10^ reported data including frozen sperm. There were 122 pregnancies from 368 ICSI cycles in the four studies that reporting fresh sperm data, and 38 pregnancies from 200 ICSI cycles in the two studies that reported data including frozen sperm. Results of the subgroup analyses revealed that testicular sperm group had significantly higher pregnancy rate than ejaculated sperm group in both subgroup of fresh sperm data (RR = 1.60, 95% CI 1.08–2.39, *P* = 0.02), and subgroup of frozen sperm and estimated data (RR = 2.42, 95% CI 1.30–4.50, *P* = 0.005). Low heterogeneity was detected in both subgroup of fresh sperm data (*I*^2^ = 19%, *P* = 0.30) and subgroup of the data including frozen sperm (*I*^2^ = 0%, *P* = 0.32) (Supplementary Figure S6).

Moreover, this study explored the influence of maternal age in pregnancy rate between the two group by using meta-regression with variable of maternal age difference. No sufficient evidence supported that maternal age difference between the two groups influenced pregnancy rates (*B* = 1.24, 95% CI 0.17–9.01, *P* = 0.69) with acceptable heterogeneity (*tau*^2^ = 0.03) (Supplementary Figure S7).

## Discussion

The present systemic review and meta-analysis showed that among patients with cryptozoospermia undergoing ICSI, the use of testicular sperm leads to higher good-quality embryo rate, implantation rate, and pregnancy rate in compared with ejaculated sperm.

Because the surgical extraction of sperm was adapted in ICSI, numerous studies have discussed how sperm origin affects clinical outcomes. These studies have yielded divergent results, and researchers have provided many probable theories to support their findings. Some evidence suggested that ejaculated sperm should lead to a better fertility outcome because of its passage through the epididymis. The epididymis plays a crucial role in the final steps of spermatogenesis, including epigenetic modification of genes^13,14^, changes in the surface proteins of spermatozoa^15^, and maturation of sperm cells^16^. Some epigenetic remodelling processes is necessary for the stability of DNA and its resistance to damage and have been reported to play a crucial role in early embryogenesis^17–21^. Alteration of these epigenomes is associated with low sperm quality, low embyro quality, and male infertility^19^. With regard to the role of the epididymis in sperm maturity, an immunostaining study demonstrated that the protein which facilitates the penetration of the zona pellucida by the sperm was found localised only on the acrosomal cap of epididymal sperm cells but not on testicular sperm cells^22^. The maturation process of sperm was also observed in IVF assays performed with spermatozoa collected at different sites along the epididymis, in which sperm retrieved from the more distal part acquired higher motility and ability to efficiently encounter the egg and its vestments^16^. Because testicular sperm does not undergo these modification processes in the epididymis, this may explain why some studies have shown higher normal fertilisation rates, implantation rates, and pregnancy rates^23–25^, and lower abortion rates in ICSI cycles using epididymal sperm than those using testicular sperm^26,27^.

However, some studies have shown no difference in outcomes of ICSI with testicular and epididymal sperm or with testicular and ejaculated sperm^28,29^. Some studies have even shown better fertility outcomes with testicular sperm than with ejaculated sperm^4,13,30^. These studies concluded that although the complex mechanisms involved in epididymal transport may be beneficial for the conventional fertilisation of oocytes, the technique of ICSI bypasses the investment and membrane of oocytes. Also, during ICSI the technician always attempts to select the sperm with highest motility and healthiest morphology through thorough examination. Therefore, the maturation of sperm is no longer a factor affecting fertilisation ability^9,29^. Damage to the sperm DNA along the genital tract might further explain why testicular sperm leads to better fertility outcomes than does ejaculated sperm. In the ICSI outcomes, studies have suggested that damage to sperm DNA may lead to impaired sperm decondensation, which reduces fertilisation rate^31,32^, produces low-quality embryos^33^, leads to implantation failure^34^, and causes recurrent pregnancy loss^35,36^. These findings raise the question of where the focus of sperm DNA damage lies during its passage through the genital tract. In a mouse model, sperm extracted from after the caudal epididymis exhibited higher chromatin aberrations and lower fertility compared with sperm extracted before the caput epididymis^37^. Furthermore, in infertile men with a high DNA fragmentation rate, testicular sperm showed less DNA damage than did ejaculated sperm^38–40^. One theory for this result is that the mature spermatozooa are tightly packed with ROS-producing immature spermatozooa while passing through the semineferous tubule and epididymis, which cause DNA damage to the mature sperm^41,42^. This theory is reasonable because spermatozoan DNA is more vulnerable to oxidation before it undergoes chromatin condensation in the epididymis. The result of our study suggests that the oxidative stress during passage through epididymis may pose more threat to ICSI outcome than the immaturity of testicular sperm.

Before our study, a previous systematic review with meta-analysis found 250 citation records, included four cohort studies and one case report, and gave a controversial conclusion that was based on incorrect pooling result of pregnancy rate from 272 ICSI cycles^12^. The previous systematic review indicated that no difference between testicular sperm group and ejaculated sperm group with forest plots for pregnancy rate (OR = 0.53, 95% CI 0.19–1.42, *P* = 0.21, *I*^2^ = 67%) and fertilisation rate (OR = 0.91, 95% CI 0.78–1.06, *P* = 0.21, *I*^2^ = 73%). These results showed moderate to high heterogeneity with less explanation and interpretation.

Our systematic review found 312 citation records, and included six cohort studies^6–11^. To conduct a reasonable quantitative synthesis, we excluded a case report^4^ that was included in the previous systematic review^12^. Our meta-analysis showed that male with cryptozoospermia in testicular sperm group had higher pregnancy rate than in ejaculated sperm group. There are two potential reasons for this result that is different from the previous systematic review. First, qualitative synthesis with different study design is a methodological difference between our study and the previous meta-analysis. The previous meta-analysis combined cohort studies with a case report, but our meta-analysis synthesized cohort studies only. Second, the previous meta-analysis^12^ reported for ejaculated sperm 9 pregnancies out of 27 attempts and for testicular sperm 5 pregnancies out of 21 attempts, while Bendickson report 9 pregnancies out 21 attempts for testicular sperm and then 5 out of 27 for testicular sperm^7^. We corrected this error in our meta-analysis.

Moreover, our meta-analysis supplemented important outcomes of good-quality embryo rate and implantation rate. We also conducted subgroup analyses to reduced confounding bias and heterogeneity. Therefore, a high heterogeneity was detected in subgroup that including estimated data and frozen sperm data (*I*^2^ = 81%, *P* = 0.006) on the result of fertilisation rate. The other subgroup that using fresh sperm data showed acceptable heterogeneity (*I*^2^ = 34%, *P* = 0.21). Other subgroup analyses revealed acceptable heterogeneity between *I*^2^ 0% to 19% in good-quality embryo rate and pregnancy rate. There is only subgroup analysis of implantation rate that showing moderate heterogeneity. The moderate heterogeneity may be affected by some limitations of this systematic review.

Although the present systematic review has much strength that was discussed above, it has some limitations. First, we cannot separate intention-to-treat and per-protocol data for meta-analysis, because of no sufficient information in the cohort studies. This may be a critical issue for all treatment recommendations. Second, we cannot solve confounding factors from risks of testicular biopsy, because of no sufficient information. Third, it is hard to deal with frozen-thawed sperm and sperm retrieval approaches perfectly, though we have tried to do subgroup analysis. We detected high heterogeneity in subgroup that including estimated data and frozen sperm data, and reduced heterogeneity from *I*^2^ = 60% to *I*^2^ = 34% in subgroup of fresh sperm data. These results raise a question regarding whether the cryopreservation of sperm affects ICSI outcomes. In our analysis, because we could not know the exact number of patients and cycles they had undergone with fresh or frozen testicular sperm in each study, we could not accurately stratify those data to observe the relations between cryopreservation and ICSI outcomes. Meanwhile, in our review of previous studies, comparing the outcomes of fresh and frozen testicular sperm, most studies focus on patients with azoospermia rather than on patients with cryptozoospermia. Therefore, this topic necessitates further investigation. Fourth, regarding to ovarian hyperstimulation protocols, evidence had showed that different ovarian hyperstimulation protocols may have influence on the ICSI outcome. A meta-analysis has shown that the pregnancy rate was found to be higher in long protocol than short or ultrashort protocol when GnRH agonist was used^43^. When comparing GnRH agonist with antagonist used in ovarian hyperstimulation, there was no difference in live birth rate but a lower incidence of ovarian hypertimulation syndrome in GnRH antagonist group^44^. For now, the available studies are not sufficient for subgroup analysis of possible influence of different ovarian hyperstimulation protocols on the ICSI outcome. This is a topic required future investigation. Fifth, the maturity and morphology of oocyte also play a role in the ICSI outcome^45^. Maternal age is an important factor for oocyte quality^46^. In the six studies included in our study, there was a trend of higher maternal age in testicular group than ejaculated group. This could be explained by the fact that non-invasive ejaculated sperm collection was usually first used in infertile couples, and testicular sperm extraction was performed only after failure with ejaculated sperm^6^. Our meta-regression analysis showed that advanced maternal age was not a significant negative predictor of pregnancy outcome. However, due to the lack of information about the intracytoplasmic or extracytoplasmic features of the metaphase II oocytes in the included six studies, the relationship between oocyte quality and ICSI outcome could not be determined. Further studies are needed for subgroup analysis of effect of oocyte quality on ICSI outcome. Sixth, Surgical recovery of testicular sperm is associated with complications including vascular injury, hematoma, infection, fibrosis or even hypogonadism^47^. This is a reason why the included studies are all cohort studies instead of prospective randomized control trial, because it would be unethical to randomly allocate patients to TESE group without first attempting sperm retrieval with ejaculation. In these studies, the numbers of failed ejaculation attempt before conducting TESE were not reported, and whether the information about possible complications after TESE was inform to the couples were also not mentioned in these studies. Because of the lack of information about the morbidity after TESE in the six studies included in our meta-analysis, the balance between risk and benefit of TESE should be handled carefully based on future studies or experts’ experience.

In summary, the present systematic review found that the use of testicular sperm led to higher good-quality embryo rate, implantation rate, and pregnancy rate than ejaculated sperm in ICSI among patients with cryptozoospermia. Although this systematic review with meta-analysis has some limitations, it still provides more informative, reliable, and clear results than previous studies. Based on existing evidence, these results may support the recommendation for male with cryptozoospermia to use testicular sperm in preference over ejaculated sperm for ICSI.

## Methods

This systematic review and meta-analysis was conducted and presented according to the Preferred Reporting Items for Systematic Reviews and Meta-Analyses guidelines^48^ (Supplementary Table 3). The protocol of this research was registered online with PROSPERO, the international prospective register of systematic reviews (CRD42017068410).

### Database and search strategy

This study was restricted to published research articles comparing the outcomes of using testicular sperm and ejaculated sperm for ICSI in the following electronic databases: PubMed, Embase, and the Cochrane Library. The systematic search did not have any restrictions of language or publication date. The final search strategy consisted of free text words and medical subject headings (MeSH and Emtree) with truncation, and it was completed on September 2017 by two authors (Y.W.H. and Y.N.K.) using relevant terms cryptozoospermia, testicular sperm extraction, and ejaculation with Boolean algebras (Supplementary Table 4).

### Eligibility criteria and evidence selection

All records were screened by two authors (Y.W.H. and Y.N.K.). Any disagreement regarding the eligibility of a study was resolved through discussion with the third author (C.C.W.). The inclusion criteria for the screening of titles and abstracts were as follows: (i) men with cryptozoospermia, and (ii) having undergone ICSI with testis sperm and ejaculated sperm. Studies were excluded if study included (i) patients who were younger than 18 years; (ii) mixed sperm retrieval methods, namely TESE and PESA in a same cohort that cannot be separated; (iii) mixed infertility conditions in a same cohort that cannot be separated.

### Quality assessment

The risk of bias in the included studies was assessed using the appraisal tool of the Newcastle-Ottawa Quality Assessment Scale for cohort studies by the two author reviewers (Y.W.H. and C.C.W.), and any disagreements were resolved by the third reviewer (Y.N.K.). Newcastle-Ottawa Quality Assessment Scale contains three aspects that including eight items associated with the risk of bias^49^. The eight items are: (i) representativeness of the exposed cohort, (ii) selection of the non-exposed cohort, (iii) ascertainment of exposure, (iv) demonstration that the outcome of interest was not present at the beginning of the study, (v) comparability of the cohorts based on the study design or analysis controlled for confounders, (vi) assessment of outcomes, (vii) length of the follow-up duration being sufficient for the outcomes to occur, and (viii) adequacy of the follow-up of the cohorts. Funnel plots, Egger’s test, and Begg’s test were conducted for publication bias assessment.

### Data extraction and analysis

In this study, two authors (Y.W.H. and Y.N.K.) extracted and checked the data independently. They identified and double-checked the number of patients, ICSI cycles, ETs, fertilisations rate, good-quality embryos, implantations, and pregnancy events. Subgroup analyses of the ICSI cycles with fresh sperm and freeze-thawed sperm were separately conducted using available data.

The RR was used for estimating dichotomous variables that were reported in the original studies. All of pooled analyses used random-effects model, and estimated *I*^2^ for heterogeneity among the included studies. The *I*^2^ value of the pooled studies was represented as the percentage of the total variability across the studies, and it defined as 75% for high heterogeneity^50^. Data were analysed by using RevMan version 5.3, and expressed RRs, 95% confidence intervals (CI), and *I*^2^ in forest plot. Funnel plot was conducted by Comprehensive Meta-Analysis version 2. Statistical significance was set at p < 0.05 for all analyses.

## Electronic supplementary material

Supplementary information
