# Supplementary material for: Testicular sperm is superior to ejaculated sperm for ICSI in cryptozoospermia: An update systematic review and meta-analysis
Source: Sci Rep. 2018 May 18;8:7874. doi: 10.1038/s41598-018-26280-0 (PMC5959851; doi:10.1038/s41598-018-26280-0)
Supplement: Supplementary file 1 — Supplementary information [file 41598_2018_26280_MOESM1_ESM.pdf]

Testicular sperm is superior to ejaculated sperm for ICSI in cryptozoospermia: An update systematic review and meta-analysis

Yi-No Kang[1*], Ya-Wen Hsiao[2*], Chien-Yu Chen[1,3,4,5], Chien-Chih Wu[1,5,6,7**]

1 Centre for Evidence-Based Medicine, Department of Education, Taipei Medical University Hospital, Taipei, Taiwan

2 School of Medicine, College of Medicine, Taipei Medical University, Taipei, Taiwan

3 Department of Anesthesiology, Taipei Medical University Hospital, Taipei, Taiwan

4 Department of Anesthesiology, School of Medicine, College of Medicine, Taipei Medical University, Taipei, Taiwan

5 Department of Education in Medicine and Humanity, School of Medicine, College of Medicine, Taipei Medical University, Taipei, Taiwan

6 Department of Urology, Taipei Medical University Hospital, Taipei, Taiwan

7 Department of Urology, School of Medicine, College of Medicine, Taipei Medical University, Taipei, Taiwan

* Co-first author

** Correspondence: Chien-Chih Wu MD, PhD

Department of Urology, Taipei Medical University Hospital, Taipei, Taiwan 252, Wu-Xing Street, Taipei 110, Taiwan

E-mail: ccwu@tmu.edu.tw

Supplementary Information

Supplementary Table 1: Oocyte quality and assessment

Supplementary Table 2: Quality assessment with Newcastle-Ottawa Quality Assessment Scale

Supplementary Table 3: PRISMA checklist

Supplementary Table 4: Database and search strategy

Supplementary Figure S1: Fertilisation rate (Funnel plot)

Supplementary Figure S2: Pregnancy rate (Funnel plot)

Supplementary Figure S3: Fertilisation rate (Subgroup analysis)

Supplementary Figure S4: Good quality embryo rate (Subgroup analysis)

Supplementary Figure S5: Implantation rate (Subgroup analysis)

Supplementary Figure S6: Pregnancy rate (Subgroup analysis)

Supplementary Figure S7: Influence of mean difference of maternal age in pregnancy rate

Item	Amirjannati et al. (2012)	Ben-Ami et al. (2013)	Bendikson et al. (2008)	Cui et al. (2016)	Hauser et al. (2011)	Ketabchi et al. (2016)
Ovarian hyperstimulation protocol	Long protocol: GnRH agonist and HMG (Menopur) or recombinent FSH (Gonal-F)	Long protocol	GnRH agonists or antagonists	Long GnRH agonist protocol	GnRH agonist (Decapeptyl) and HMG (Menogon) or recombinant FSH (Gonal F)	Modified superlong protocol with GnRH agonist and HMG
Ovulation method	10000IU HCG (Choriomon)	NR	HCG	4000-10000U HCG	HCG (Pregnyl 10,000 IU)	NR
Lag time from ovulation trigger to oocyte aspiration	NR	NR	Approximately 35-36 hours	36 hours	34-35 hours	NR
Assessment of oocyte maturity	GV, MI,MII were assessed, reported as number of MII oocytes	Number of MII oocytes	Number of MII oocytes	NR	Number of MII oocytes	NR
Support after embryo transfer	NR	IM injection of HCG or 600mg vaginally administered micronized P (Utrogestan)	Methylprednisolone 16 mg daily and tetracycline 250 mg every 6 hours were administered for 4 days starting from oocyte retrieval. Progesterone 25 to 50 mg per day were administered intramuscularly until pregnancy.	NR	NR	NR
Continued						

Item	Amirjannati et al. (2012)	Ben-Ami et al. (2013)	Bendikson et al. (2008)	Cui et al. (2016)	Hauser et al. (2011)	Ketabchi et al. (2016)
Assessment of embryo quality	The embryos were graded 72 h following fertilisation according to their morphology, fragmentation quantity, blastomeres number and symmetry status, classified as grade A~C.	The embryos were graded at day2 and day3 following fertilisation according to their mean number of blastomeres and morphology score.	The embryos were assessed by the average number of blastomeres and the proportion of fragmentation, presented as the number of embryos replaced.	The embryos were graded according to the size and symmetry status of blastomeres, and the fragmentation quantity of nuclear debris.	The embryos were graded at day2 and day3 following fertilisation according to the number of blastomeres, the degree of fragmentation, and the extent of compaction. High quality embryo defined as fragmentation < 20%.	Embryonic morphologic assessment was performed on day 2 after the ovum pick-up and then on day 3. The embryos at the eight-cell stage with less than 20% fragmentation were described as good quality embryos.

Supplementary Table 1 Oocyte recovery and assessment of the included studies. GnRH, gonadotropin-releasing hormone; HMG, human menopausal gonadotrophin; HCG, human chorionic gonadotropin; FSH, follicle-stimulating hormone; MII, metaphase II oocyte; 2PN: 2-pronuclear zygote; NR, not reported.

	Amirjannati et al. (2012)	Ben-Ami et al. (2013)	Bendikson et al. (2008)	Cui et al. (2016)	Hauser et al. (2011)	Ketabchi et al. (2016)
**Selection**						
Representativeness of the exposed cohort	★	★	★	★	★	★
Selection of the non-exposed cohort	★	★	★	★	★	★
Ascertainment of exposure	★	★	★	★	★	★
Demonstration that outcome of interest was not present at start of study	★	★	★	★	★	★
**Comparability**						
Comparability of cohorts on the basis of the design or analysis controlled for confounders	★	★	★	★★	★★	No detail
**Outcome**						
Assessment of outcome	★	★	★	★	★	★
Was follow-up long enough for outcomes to occur	★	★	★	★	★	★
Adequacy of follow-up of cohorts	★	★	★	★	★	★

Supplementary Table 2 Quality assessment with Newcastle-Ottawa Quality Assessment Scale.

PRISMA 2009 Checklist

Section/topic	#	Checklist item	Reported on page #
**TITLE**			
Title	1	Identify the report as a systematic review, meta-analysis, or both.	1
**ABSTRACT**			
Structured summary	2	Provide a structured summary including, as applicable: background; objectives; data sources; study eligibility criteria, participants, and interventions; study appraisal and synthesis methods; results; limitations; conclusions and implications of key findings; systematic review registration number.	2
**INTRODUCTION**			
Rationale	3	Describe the rationale for the review in the context of what is already known.	3
Objectives	4	Provide an explicit statement of questions being addressed with reference to participants, interventions, comparisons, outcomes, and study design (PICOS).	3
**METHODS**			
Protocol and registration	5	Indicate if a review protocol exists, if and where it can be accessed (e.g., Web address), and, if available, provide registration information including registration number.	15
Eligibility criteria	6	Specify study characteristics (e.g., PICOS, length of follow-up) and report characteristics (e.g., years considered, language, publication status) used as criteria for eligibility, giving rationale.	16
Information sources	7	Describe all information sources (e.g., databases with dates of coverage, contact with study authors to identify additional studies) in the search and date last searched.	16
Search	8	Present full electronic search strategy for at least one database, including any limits used, such that it could be repeated.	16
Study selection	9	State the process for selecting studies (i.e., screening, eligibility, included in systematic review, and, if applicable, included in the meta-analysis).	16
Data collection process	10	Describe method of data extraction from reports (e.g., piloted forms, independently, in duplicate) and any processes for obtaining and confirming data from investigators.	17
Data items	11	List and define all variables for which data were sought (e.g., PICOS, funding sources) and any assumptions and simplifications made.	17
Risk of bias in individual studies	12	Describe methods used for assessing risk of bias of individual studies (including specification of whether this was done at the study or outcome level), and how this information is to be used in any data synthesis.	16-17
Summary measures	13	State the principal summary measures (e.g., risk ratio, difference in means).	17
Synthesis of results	14	Describe the methods of handling data and combining results of studies, if done, including measures of consistency (e.g., I^2) for each meta-analysis.	17

PRISMA 2009 Checklist

Section/topic	#	Checklist item	Reported on page #
Risk of bias across studies	15	Specify any assessment of risk of bias that may affect the cumulative evidence (e.g., publication bias, selective reporting within studies).	17
Additional analyses	16	Describe methods of additional analyses (e.g., sensitivity or subgroup analyses, meta-regression), if done, indicating which were pre-specified.	17
**RESULTS**			
Study selection	17	Give numbers of studies screened, assessed for eligibility, and included in the review, with reasons for exclusions at each stage, ideally with a flow diagram.	4
Study characteristics	18	For each study, present characteristics for which data were extracted (e.g., study size, PICOS, follow-up period) and provide the citations.	4-5
Risk of bias within studies	19	Present data on risk of bias of each study and, if available, any outcome level assessment (see item 12).	5
Results of individual studies	20	For all outcomes considered (benefits or harms), present, for each study: (a) simple summary data for each intervention group (b) effect estimates and confidence intervals, ideally with a forest plot.	5-9
Synthesis of results	21	Present results of each meta-analysis done, including confidence intervals and measures of consistency.	5-9
Risk of bias across studies	22	Present results of any assessment of risk of bias across studies (see Item 15).	5, 7
Additional analysis	23	Give results of additional analyses, if done (e.g., sensitivity or subgroup analyses, meta-regression [see Item 16]).	8-9
**DISCUSSION**			
Summary of evidence	24	Summarize the main findings including the strength of evidence for each main outcome; consider their relevance to key groups (e.g., healthcare providers, users, and policy makers).	10
Limitations	25	Discuss limitations at study and outcome level (e.g., risk of bias), and at review-level (e.g., incomplete retrieval of identified research, reporting bias).	13-15
Conclusions	26	Provide a general interpretation of the results in the context of other evidence, and implications for future research.	15
**FUNDING**			
Funding	27	Describe sources of funding for the systematic review and other support (e.g., supply of data); role of funders for the systematic review.	21

From: Moher D, Liberati A, Tetzlaff J, Altman DG, The PRISMA Group (2009). Preferred Reporting Items for Systematic Reviews and Meta-Analyses: The PRISMA Statement. PLoS Med 6(6): e1000097. doi:10.1371/journal.pmed1000097

For more information, visit: **www.prisma-statement.org**.

Database	Syntax	Returns
Cochrane	'cryptozoospermi* OR cryptozoospermia OR virtual azoospermia OR cryptozoospermic' and 'testis OR testicular OR testic* OR testicular sperm extraction OR TESE' and 'ejaculation OR ejaculated OR ejaculat*' and 'icsi OR intracytoplasmic sperm injection OR in vitro fertilization in Trials'	5
Embase	(cryptozoospermi* OR 'cryptozoospermia'/exp OR 'cryptozoospermia' OR cryptozoospermic OR 'virtual azoospermia') AND (icsi OR 'intracytoplasmic sperm injection'/exp OR 'icsi' OR 'injection, intracytoplasmic sperm' OR 'intracytoplasmic sperm injection' OR 'sperm injections, intracytoplasmic' OR 'in vitro fertilization'/exp OR 'ivf (in vitro fertilization)' OR 'extracorporeal fertilization' OR 'fertilization in vitro' OR 'in vitro fertilisation' OR 'in vitro fertilization' OR 'testtube baby') AND ('testis'/exp OR 'left testicle' OR 'left testis' OR 'right testicle' OR 'right testis' OR 'testicle' OR 'testis' OR testicular OR testic* OR 'testicular sperm extraction'/exp OR 'tese' OR 'testicular sperm extraction' OR 'testis sperm extraction' OR tese) AND (ejaculated OR 'ejaculation'/exp OR 'ejaculation' OR 'seminal discharge' OR 'sperm release' OR ejaculat*)	53
Ovid MEDLINE	1 (cryptozoospermi* or cryptozoospermia or virtual azoospermia or cryptozoospermic).af. (139)<br>2 (testis or testicular or testic* or testicular sperm extraction or TESE).af. (67809)<br>3 (ejaculation or ejaculated or ejaculat*).af. (18121)<br>4 (icsi or intracytoplasmic sperm injection or in vitro fertilization).af. (23183)<br>5 1 and 2 and 3 and 4 (73)	73
PubMed	(cryptozoospermi* OR cryptozoospermia OR virtual azoospermia OR cryptozoospermic) AND (testis OR testicular OR testic* OR testicular sperm extraction OR TESE) AND (ejaculation OR ejaculated OR ejaculat*) AND (icsi OR intracytoplasmic sperm injection OR in vitro fertilization)	25
Continued		

Database	Syntax	Returns
ScienceDirect	(cryptozoospermi* OR cryptozoospermia OR virtual azoospermia OR cryptozoospermic) AND (testis OR testicular OR testic* OR testicular sperm extraction OR TESE) AND (ejaculation OR ejaculated OR ejaculat*) AND (icsi OR intracytoplasmic sperm injection OR in vitro fertilization)	49
Scopus	(cryptozoospermi* OR cryptozoospermia OR virtual azoospermia OR cryptozoospermic) AND (testis OR testicular OR testic* OR testicular sperm extraction OR TESE) AND (ejaculation OR ejaculated OR ejaculat*) AND (icsi OR intracytoplasmic sperm injection OR in vitro fertilization)	70
Web of Science	TOPIC: ((cryptozoospermi* OR cryptozoospermia OR virtual azoospermia OR cryptozoospermic)) AND TOPIC: ((testis OR testicular OR testic* OR testicular sperm extraction OR TESE)) AND TOPIC: ((icsi OR intracytoplasmic sperm injection OR in vitro fertilization)) AND TOPIC: ((ejaculation OR ejaculated OR ejaculat*))	36

Supplementary Table 4 Database and search strategy.

Supplementary figure

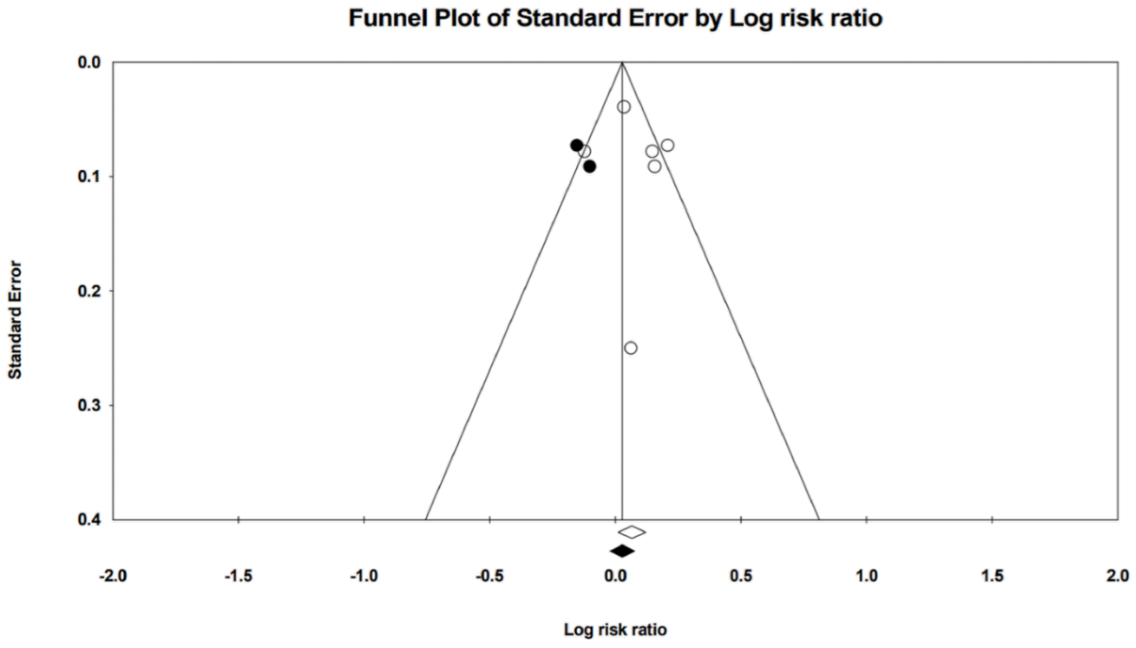

Supplementary Figure S1: Fertilisation rate (Funnel plot)

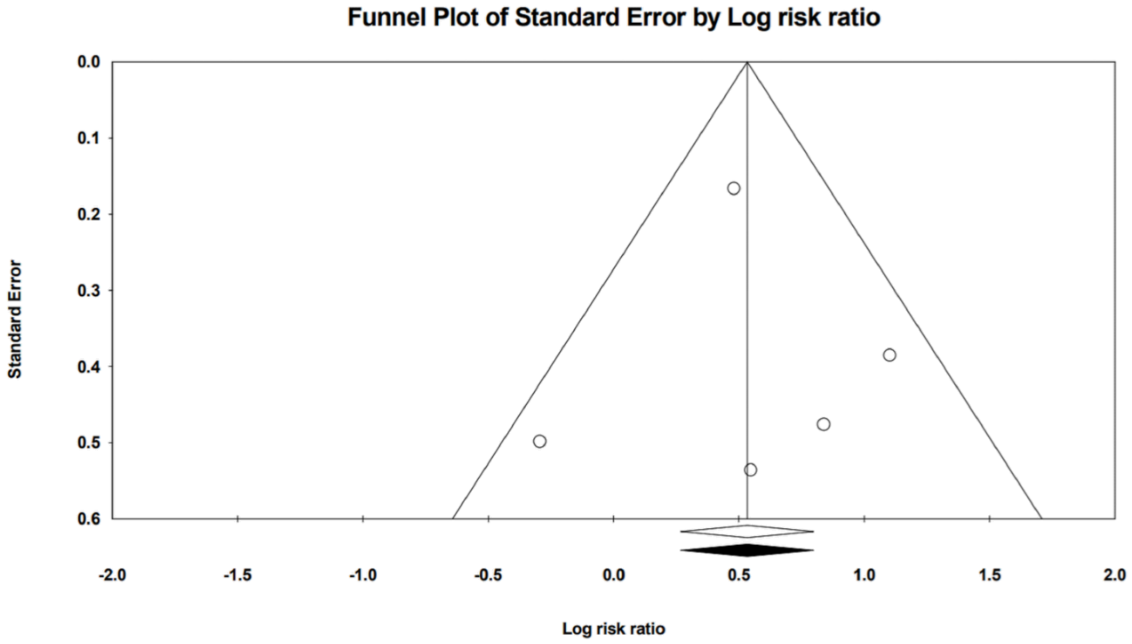

Supplementary Figure S2: Pregnancy rate (Funnel plot)

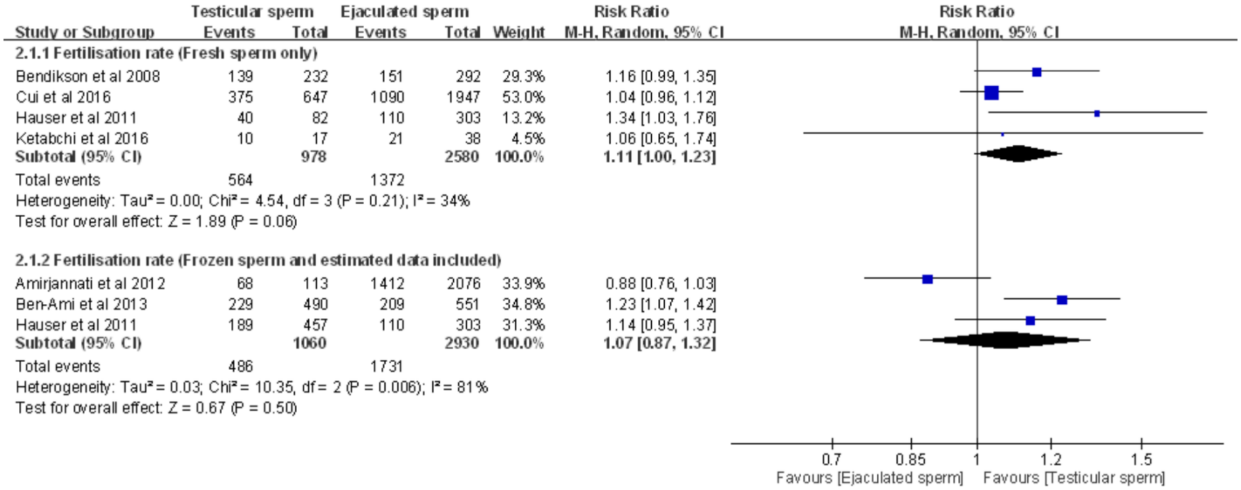

Supplementary Figure S3: Fertilisation rate (Subgroup analysis)

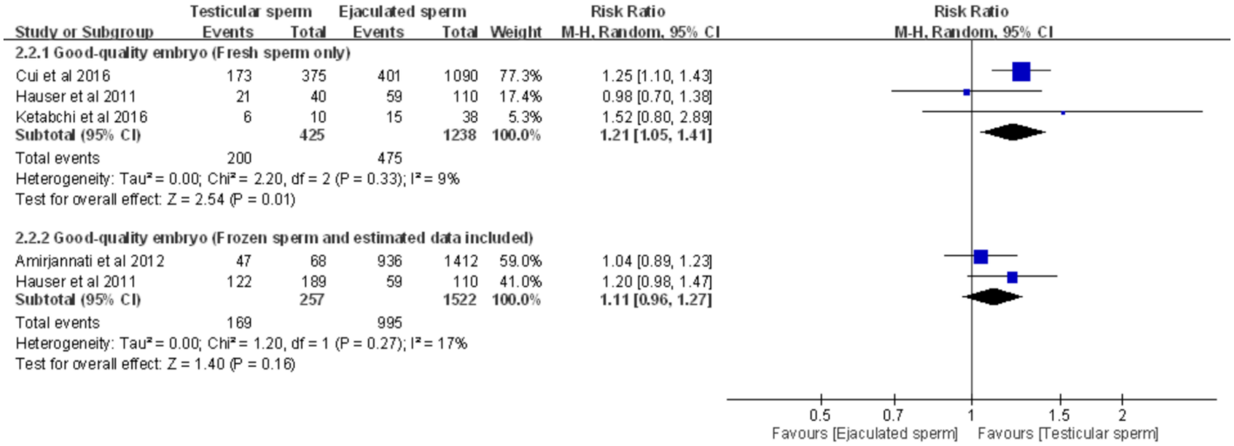

Supplementary Figure S4: Good quality embryo rate (Subgroup analysis)

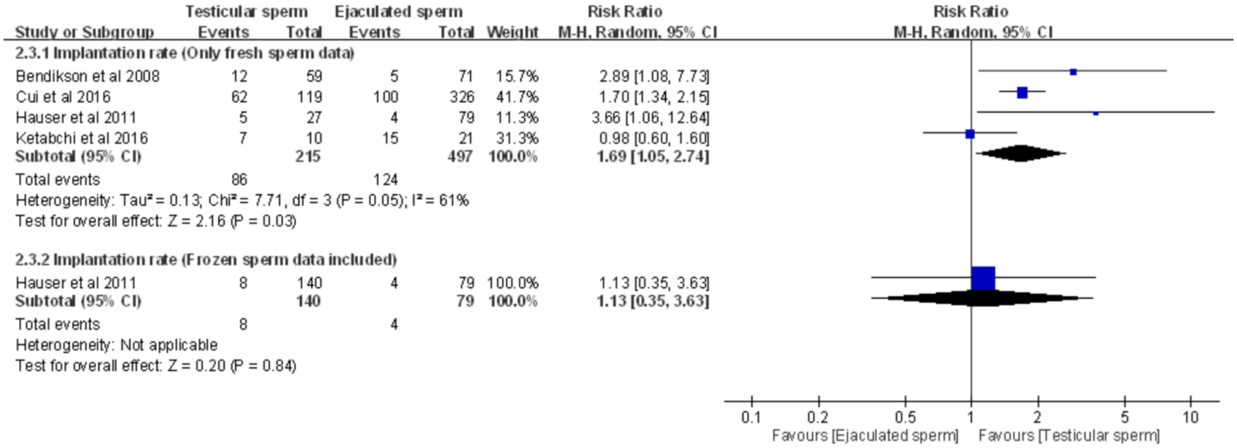

Supplementary Figure S5: Implantation rate (Subgroup analysis)

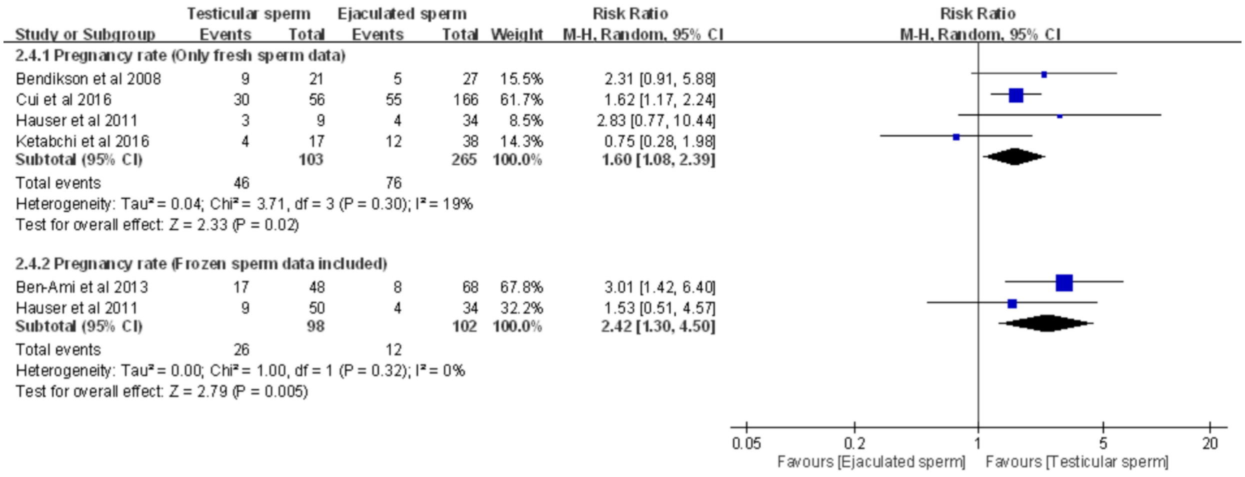

Supplementary Figure S6: Pregnancy rate (Subgroup analysis)

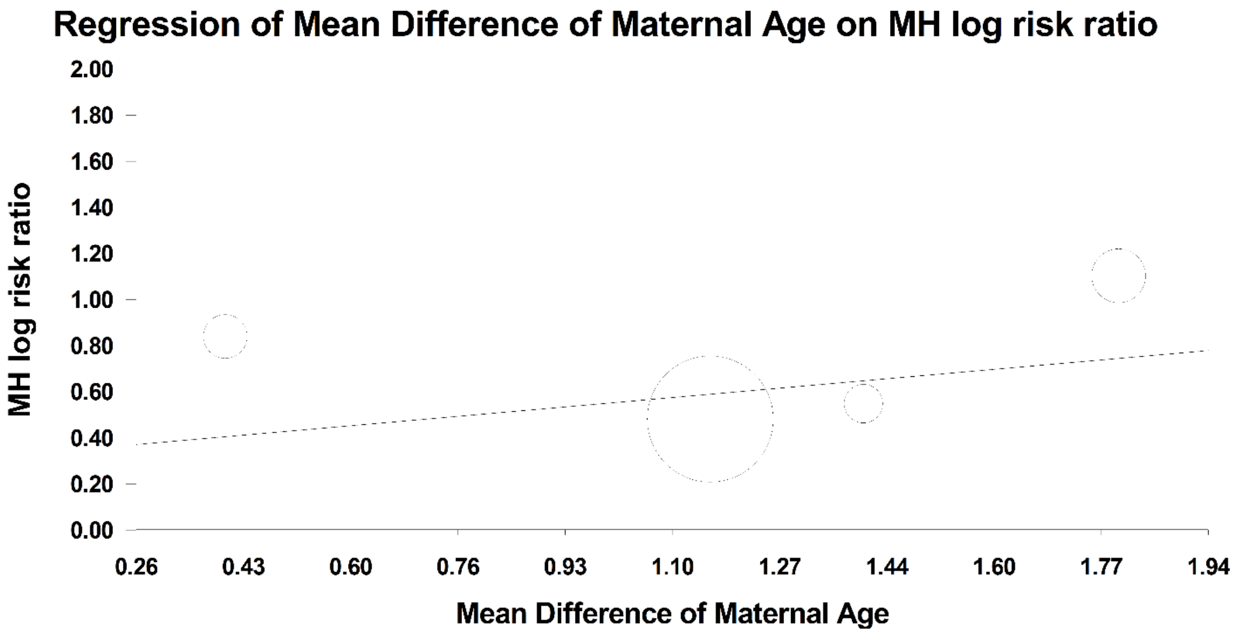

Supplementary Figure S7: Influence of mean difference of maternal age in pregnancy rate
